# A Rare Case of Acute Bacterial Native Valve Endocarditis Caused by Streptococcus thoraltensis

**DOI:** 10.7759/cureus.70934

**Published:** 2024-10-06

**Authors:** Shaun Abid, Anton Stolear, Maxim Dulgher, Samdish Sethi, Stuart Zarich

**Affiliations:** 1 Internal Medicine, Yale University, Bridgeport Hospital, Bridgeport, USA; 2 Cardiology, Yale University, Bridgeport Hospital, Bridgeport, USA; 3 Internal Medicine, Nuvance Health, Norwalk Hospital, Norwalk, USA; 4 Internal Medicine and Cardiology, Yale University, Bridgeport Hospital, Bridgeport, USA; 5 Cardiology, Bridgeport Hospital, Bridgeport, USA

**Keywords:** cardiology research, drug use associated infective endocarditis, emerging pathogen, infective endocarditis, streptococcus thoraltensis, tricuspid valve endocarditis

## Abstract

This report presents a rare case of acute bacterial native valve endocarditis caused by *Streptococcus thoraltensis* in a 57-year-old male with a history of intravenous drug use. The patient presented with chest pain, productive cough, and diarrhea, with clinical evaluation revealing atrial flutter, pulmonary embolism, and a large tricuspid valve vegetation. Blood cultures confirmed *Streptococcus thoraltensis*, an organism rarely implicated in human infections. The patient’s prior work at an animal shelter, with direct handling of rabbits, suggests a possible zoonotic transmission of the infection.

Treatment with intravenous ceftriaxone resulted in partial clinical improvement, but the patient was lost to follow-up. This case highlights the importance of considering rare pathogens in endocarditis, particularly in patients with exposure to animals or intravenous drug use. It underscores the need for thorough patient history in guiding diagnosis and treatment. It adds to the limited literature on *Streptococcus thoraltensis* as a human pathogen, emphasizing the need for increased awareness and documentation.

## Introduction

*Streptococcus thoraltensis* is a gram-positive coccus first described by Devriese et al. in 1997, who isolated it from the vaginal fluids and intestines of pigs [1]. This species has subsequently been isolated from rabbit feces [2], the human oral cavity [3], the placenta [4], and in only five reported cases, the human blood [5]. To our knowledge, only one other case of native valve bacterial endocarditis due to this bacterium has ever been reported [5], as well as a single case of prosthetic valve bacterial endocarditis [6]. Intravenous drug use (IVDU) is a strong risk factor for infective endocarditis. The organisms most frequently reported to cause bacterial endocarditis in patients who inject intravenous recreational drugs are *Staphylococcus aureus*, followed by Streptococcus and Enterococci [7].

## Case presentation

A 57-year-old, previously healthy, immune-competent animal shelter worker who was admitted to intravenous recreational drug use presented with a two-week history of progressively worsening chest pain, cough, and diarrhea. At presentation, he was afebrile, tachycardic (170 bpm), normotensive (109/75 mmHg), saturating 100% on room air, and complained of severe chest pain (10/10). The chest pain was described as substernal, pleuritic, radiating to the left shoulder, and associated with nausea and vomiting. The cough was productive with yellow phlegm and had been progressively worsening. Diarrhea had also been present for two weeks, with at least two episodes per day, and was associated with an unintentional 20-pound weight loss.

In the emergency room, the patient was noted to be in atrial flutter with a ventricular response of 170 bpm, which did not respond to intravenous diltiazem. He developed low blood pressure despite having received two liters of normal saline. The rhythm was subsequently electrically cardioverted to sinus rhythm. He was then transferred to the intensive care unit (ICU) and placed on intravenous pressors.

**Figure 1:**
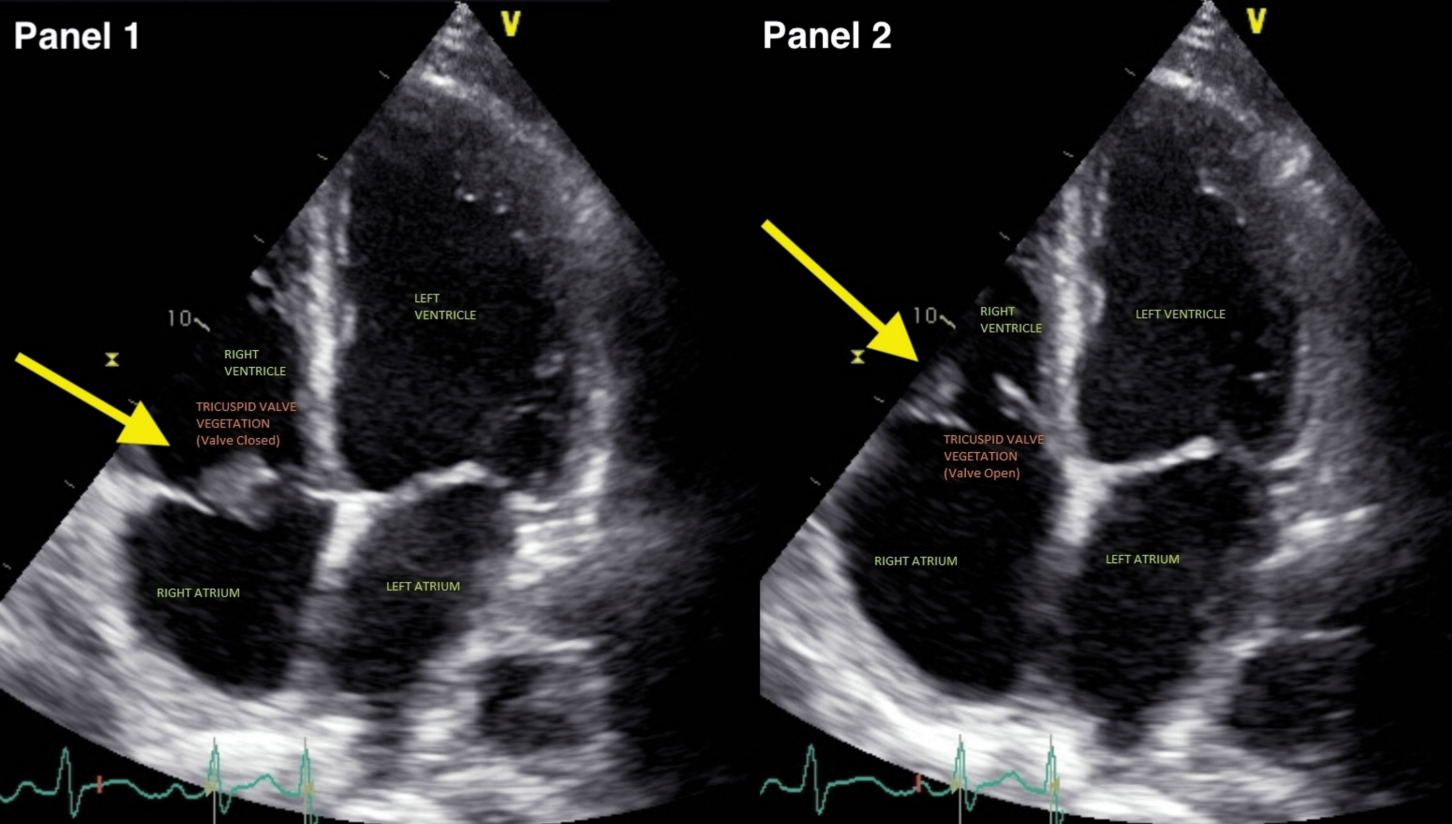
Echocardiogram showing large tricuspid valve vegetation in a patient with Streptococcus thoraltensis endocarditis. This figure contains two panels. Panel 1 shows the echocardiogram with the tricuspid valve vegetation visible when the valve is closed. Panel 2 shows the vegetation with the valve open, highlighting the dynamic nature of the vegetation on the tricuspid valve.

Admission laboratory data was significant for leukocytosis of 22,700 per cubic mm with a left shift, hyponatremia (129 mmol/L), and a normal troponin-I level. Chest CT revealed a left upper lobe pulmonary embolism and mild right-sided airspace disease attributed to pneumonia or atelectasis. An echocardiogram revealed a large vegetation on the tricuspid valve, moderate tricuspid regurgitation, borderline pulmonary hypertension, and severe left ventricular systolic dysfunction with an ejection fraction of 25% and severe global hypokinesis (Figure 1). Initial diagnoses included acute sepsis, community-acquired pneumonia, septic emboli, infective endocarditis, and possible acute coronary syndrome.

**Figure 2:**
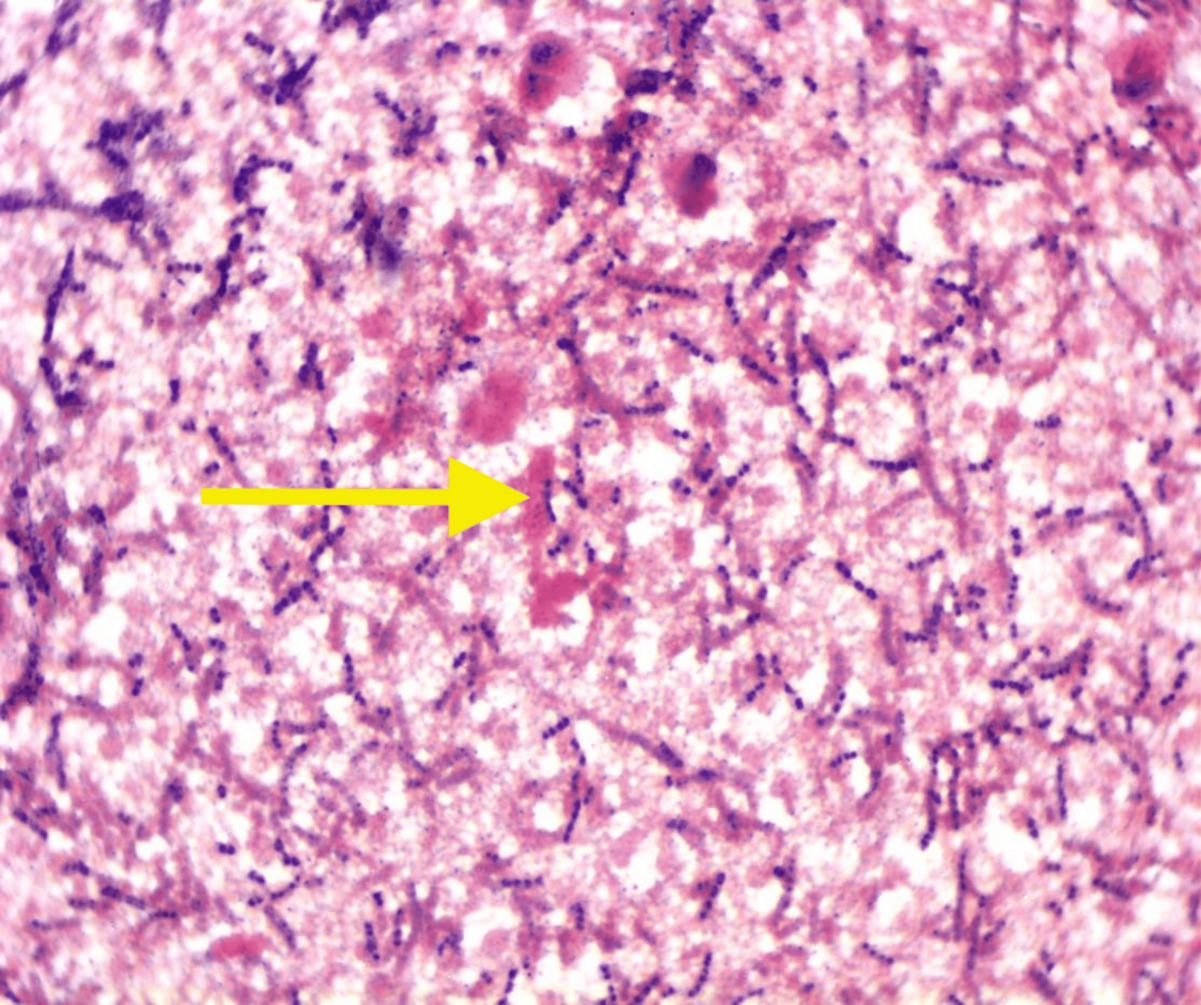
Histological image of gram-positive cocci seen in chains (H&E staining). This figure shows a histological section stained with hematoxylin and eosin (H&E), highlighting gram-positive cocci arranged in chains, characteristic of Streptococcus species.

**Figure 3:**
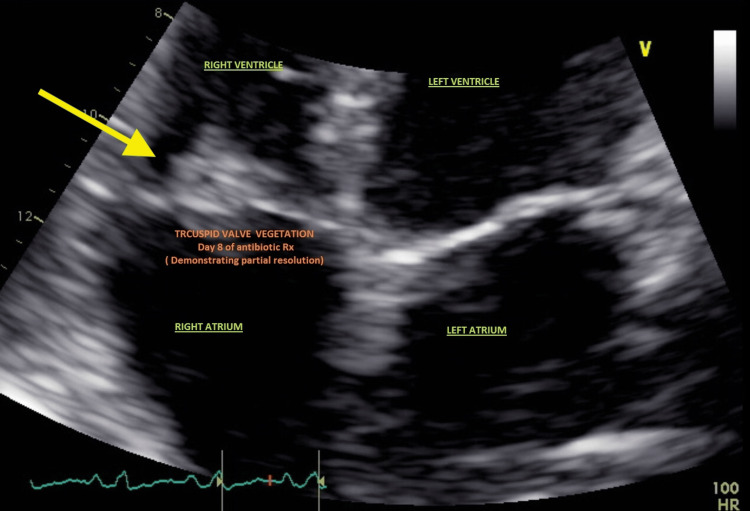
Echocardiogram demonstrating partial resolution of tricuspid valve vegetation after eight days of antibiotic therapy.

Prior to culture growth, antibiotic management was initially started with vancomycin alongside piperacillin and tazobactam (Zosyn). Zosyn was subsequently discontinued, and vancomycin was continued while adding nafcillin and gentamicin. Preliminary peripheral blood cultures grew gram-positive Streptococcus in three out of four bottles (Figure 2), which was sensitive to ampicillin, penicillin G, ceftriaxone, clindamycin, erythromycin, and vancomycin. Seventy-two hours after admission, the pathogen was identified as *Streptococcus thoraltensis* with 93% specificity using the automated system Vitek 2 Technology. Sensitivity-based antibiotic therapy was narrowed to include only IV ceftriaxone. Repeat blood cultures documented clearance of the bacteremia. A 2D echocardiogram performed eight days after admission demonstrated partial resolution of the tricuspid vegetation (Figure 3).

After identifying the organism, we conducted a thorough patient history. He immigrated from the Dominican Republic in the 1980s and had used IV heroin for over 20 years. At the time of presentation, he was working as a handyman. He lived at home with his girlfriend and denied having any pets. He denied having done any work on a farm or having any recent contact with pigs, rabbits, or cows. He did recall, however, that about a year and a half prior to admission, he had worked at an animal shelter for about 16 months. His contact was mostly with domestic animals. He recalled having cared for some rabbits for about three months. He had been responsible for feeding the rabbits and cleaning their crates. He reported that he did not always wear gloves when caring for the rabbits. He was also using IV heroin during this time period. He did not remember if he always cleaned his injection site, which was usually in his arms or legs. This social and work history suggested the hypothesis that his skin had become colonized with* S. thoraltensis* during his time working with the rabbits. IV injection of heroin through incompletely cleaned skin may have introduced the bacteria into the bloodstream, leading to endocarditis. *S. thoraltensis* was identified with 93% specificity using Vitek 2 Technology. While specific 16s rRNA typing would have been ideal, it was not performed. Nonetheless, *S. thoraltensis* was isolated from three out of four blood culture bottles, strongly suggesting it is the pathogen. Antibiotics were successful based on susceptibility testing, but the patient was lost to follow-up, precluding confirmation of clinical cure or the fate of the tricuspid valve vegetation.

## Discussion

*Streptococcus thoraltensis* was first reported to cause human infection in 2014, identified in a case of periodontitis through subgingival plaque sampling [3]. To date, documented cases of this bacterium as a human pathogen remain exceedingly rare. After conducting an extensive review of the available literature, including searches through Google and PubMed, this case appears to be only the second reported instance of *S. thoraltensis* causing native valve bacterial endocarditis, highlighting the rare but possible role of zoonotic transmission in human disease.

The patient’s occupational exposure to rabbits while working at an animal shelter is a key detail that suggests a plausible transmission route for this infection. Given that *S. thoraltensis* is typically isolated from animal flora, particularly from rabbits and pigs, the patient’s direct handling of rabbits without consistent use of protective gloves raises the likelihood of skin colonization with this pathogen. The subsequent introduction of *S. thoraltensis* into the bloodstream, likely through intravenous drug use, provides a reasonable explanation for the development of endocarditis in this case. This highlights the significance of thorough social and occupational histories in clinical practice, particularly for patients presenting with unusual infections or those with complex lifestyles, such as intravenous drug users.

The identification of *S. thoraltensis* in this patient was achieved using the automated Vitek 2 system, with a 93% specificity rate. While more advanced methods, such as 16S rRNA gene sequencing, would have provided a higher degree of accuracy in confirming the pathogen, the growth of *S. thoraltensis* in three out of four peripheral blood culture bottles strongly suggests that it was the causative agent of the endocarditis. This case highlights the potential for *S. thoraltensis* to act as an opportunistic pathogen, particularly in individuals with certain risk factors, such as intravenous drug use and occupational exposure to animals.

This case also emphasizes the importance of maintaining vigilance for uncommon or rare pathogens in patients with infective endocarditis, particularly those with relevant exposure risks, such as working closely with animals. Healthcare providers should remain aware of the potential for zoonotic infections, especially when treating populations at risk of contracting rare pathogens, such as veterinarians, farmers, or animal shelter workers. Additionally, the co-occurrence of intravenous drug use presents further complications, as the introduction of pathogens via contaminated skin or needles remains a serious concern in this population. This underscores the need for meticulous hygiene practices among intravenous drug users, as even rare organisms like *S. thoraltensis* can lead to severe infections such as endocarditis.

The patient, in this case, was treated with intravenous ceftriaxone based on susceptibility testing, and initial clinical improvement was noted. Unfortunately, the patient was lost to follow-up, which precludes definitive conclusions regarding the resolution of the infection or the fate of the tricuspid valve vegetation. This underscores a significant challenge in managing patients with intravenous drug use, as they are often at higher risk for poor compliance with long-term treatment and follow-up care.

## Conclusions

This case adds to the limited but growing body of knowledge surrounding *Streptococcus thoraltensis* as a human pathogen. Its rarity highlights the need for early recognition and appropriate treatment, particularly in patients with specific risk factors such as occupational exposure to animals or intravenous drug use. Continued documentation and reporting of such cases are essential to expanding our understanding of the pathogenic mechanisms, epidemiology, and optimal treatment strategies for *S. thoraltensis* infections. Clinicians must remain vigilant in considering zoonotic pathogens in the differential diagnosis of infective endocarditis, especially when dealing with unusual or rare organisms. Awareness of potential transmission from animals to humans in high-risk populations is critical for timely diagnosis and intervention.

Further research is also needed to define better the pathogenic pathways of *S. thoraltensis* and its reservoirs in both human and animal populations. Ongoing case reporting will help build a more comprehensive medical knowledge base, ultimately aiding in the development of effective preventive measures, diagnostic tools, and treatment protocols for this rare infection.
